# Distinct gut microbiome profiles in Korean systemic lupus erythematosus patients

**DOI:** 10.1186/s12967-025-07438-7

**Published:** 2025-12-23

**Authors:** Youngjae Park, Jiwon Yang, Hahee Son, Min-Jung Park, Su-Jin Moon, Ki-Jo Kim, Seung-Ki Kwok

**Affiliations:** 1 Division of Rheumatology, Department of Internal Medicine, Seoul St. Mary’s Hospital, College of Medicine, The Catholic University of Korea, 222 Banpo-daero, Seocho-gu, Seoul, 06591 Republic of Korea; 2 Division of Rheumatology, Department of Internal Medicine, Yeouido St. Mary’s Hospital, College of Medicine, The Catholic University of Korea, Seoul, Republic of Korea; 3 The Rheumatism Research Center, Catholic Research Institute of Medical Science, The Catholic University of Korea, Seoul, Republic of Korea; 4 Division of Rheumatology, Department of Internal Medicine, St. Vincent’s Hospital, College of Medicine, The Catholic University of Korea, Seoul, Republic of Korea

## Abstract

**Background:**

Systemic lupus erythematosus (SLE) is an autoimmune disease associated with systemic inflammation and multi-organ involvement. Emerging evidence suggests that gut microbiota dysbiosis may contribute to its immunopathogenesis.

**Objective:**

This study aimed to characterize gut microbial composition and diversity in Korean SLE patients and evaluate associations with clinical features.

**Methods:**

Fecal samples from 157 SLE patients and 50 healthy controls (HC) were analyzed using 16S rRNA gene sequencing. Alpha and beta diversity metrics were assessed, and taxonomic differences were analyzed. Subgroup comparisons were conducted based on lupus nephritis (LN) status and disease activity. Functional predictions were inferred using PICRUSt2.

**Results:**

SLE patients exhibited significantly reduced microbial richness (Chao1, ACE, Fisher indices), while evenness (Shannon, Simpson) was preserved. Beta diversity analysis revealed distinct clustering between SLE and HC groups. SLE was characterized by enrichment of *Bacteroides*, *Streptococcus*, and *Veillonella*, and depletion of *Collinsella*, *Ruminococcus*, and *Bifidobacterium*. LEfSe identified several discriminatory taxa. However, no significant microbial differences were observed between LN-positive and LN-negative groups or between high and low disease activity groups. Functional prediction revealed minimal differences in microbial pathways between groups.

**Conclusion:**

These findings highlight distinct gut microbial alterations in Korean SLE patients and support the potential utility of microbiome profiles as diagnostic biomarkers or therapeutics.

**Supplementary information:**

The online version contains supplementary material available at 10.1186/s12967-025-07438-7.

## Introduction

Systemic lupus erythematosus (SLE) is a chronic, systemic autoimmune disease characterized by the production of diverse autoantibodies and immune complexes, resulting in inflammation and damage to multiple organ systems. The clinical heterogeneity of SLE, ranging from mild cutaneous involvement to life-threatening renal, neurologic, or cardiovascular manifestations, reflects the complexity of its underlying immunopathogenesis [1]. Although genetic predisposition is a well-established factor in SLE, concordance rates among monozygotic twins remain below 60%, underscoring the critical role of environmental factors in disease development and progression [2].

Among these environmental influences, the gut microbiota has recently emerged as a pivotal contributor to autoimmune diseases, including SLE [3, 4]. The human gut microbiome is composed of trillions of microorganisms that participate in metabolic, immunological, and protective functions. Dysbiosis, defined as an imbalance in microbial community structure and function, has been associated with a range of chronic conditions such as inflammatory bowel disease, rheumatoid arthritis, and type 1 diabetes [5]. In SLE, accumulating evidence from human and animal studies suggests that gut dysbiosis may not only reflect disease activity but also actively drive autoimmunity [3, 6]. Early studies demonstrated a consistent reduction in alpha diversity and alterations in the Firmicutes to Bacteroidetes ratio in the gut microbiota of SLE patients [6, 7]. Certain bacterial taxa, such as *Ruminococcus gnavus*, *Eggerthella*, and *Prevotella*, have been repeatedly identified as expanded in SLE and linked to increased disease activity, renal involvement, and systemic inflammation [3, 6, 8]. Mechanistically, these pathobionts are proposed to compromise gut epithelial barrier integrity, allowing translocation of microbial products that stimulate autoreactive T and B cells through molecular mimicry and antigenic stimulation [3, 6].

Recent advances in metagenomic and metabolomic approaches have further highlighted the interplay between microbiota-derived metabolites and host immunity. Microbial fermentation products such as short-chain fatty acids (SCFA) influence regulatory T cell differentiation and mucosal tolerance, whereas bacterial endotoxins can activate pattern recognition receptors such as TLR4 and NLRs, promoting systemic inflammation [5]. A study by Chen et al. demonstrated that SLE-enriched microbial species could generate proinflammatory peptides that mimic host autoantigens, inducing systemic immune activation even in the absence of overt infection [3]. Although cross-sectional case-control studies in various populations such as Chinese, Spanish, French, and American have consistently reported gut microbial alterations in SLE, inter-cohort differences exist, potentially influenced by ethnicity, diet, geography, and treatment status [4, 6, 7]. Moreover, the clinical relevance of such microbial alterations, particularly their association with organ-specific involvement such as lupus nephritis (LN) or disease activity, remains uncertain [3, 4].

In this study, we aimed to comprehensively characterize the gut microbiota of 157 Korean patients with SLE using 16S rRNA gene sequencing and evaluate differences in microbial diversity and composition compared to 50 healthy controls (HC). Additionally, we investigated whether gut microbiota profiles differ according to LN status or disease activity level within the SLE cohort. Our findings contribute to a growing body of literature on SLE-associated dysbiosis and provide insights into its population-specific features and potential as a diagnostic or therapeutic biomarker.

## Materials and methods

### Study population and sample collection

This study included 157 patients diagnosed with SLE based on the 2019 European League Against Rheumatism/American College of Rheumatology (ACR) classification criteria [9]. Patients were prospectively recruited from Seoul St. Mary’s Hospital and Yeouido St. Mary’s Hospital, Seoul, Republic of Korea. Fifty age- and sex-matched healthy individuals with no history of autoimmune or gastrointestinal diseases and no recent antibiotic use (within 3 months) were enrolled as HC. Demographic and clinical data, including age, sex, disease duration, medication use, presence of LN, and clinical parameters such as Systemic Lupus International Collaborating Clinics (SLICC)/ACR damage indices and SLE Disease Activity Index (SLEDAI) scores, were recorded at the time of sample collection [10, 11]. Dietary and lifestyle information (smoking status and amount, coffee and alcohol consumption) was collected using a standardized questionnaire. However, these data were not incorporated as covariates in the analysis because no dietary restrictions were imposed prior to stool sampling and the questionnaire results were primarily descriptive. Medication information, including glucocorticoids, hydroxychloroquine, gastrointestinal agents, and belimumab, was collected for all SLE patients. Fresh fecal samples were obtained from participants using sterile containers and immediately stored at −80 °C until DNA extraction.

### Dna extraction, PCR amplification, and sequencing

Genomic DNA was extracted using the Maxwell® RSC PureFood GMO and Authentication Kit (Promega, Madison, WI, USA), following the manufacturer’s protocols. All samples were processed using the same extraction protocol. Fecal samples were submitted sequentially in small numbers and were extracted and sequenced in a randomized and blinded manner, ensuring that sample processing order was unrelated to disease status. For bacterial profiling, the V3–V4 hypervariable regions of the 16S rRNA gene were amplified using fusion primers 341F (5’-CCTACGGGNGGCWGCAG-3’) and 805R (5’-GACTACHVGGGTATCTAATCC-3’). Fusion primers included Nextera adapters and unique barcodes for multiplexing. PCR amplification was performed with the following conditions: 95 °C for 3 min, followed by 25 cycles of 95 °C for 30 sec, 55 °C for 30 sec, and 72 °C for 30 sec, with a final extension at 72 °C for 5 min. PCR products were verified by 1% agarose gel electrophoresis, purified using magnetic beads, and pooled in equimolar concentrations. Short fragments were removed using the ProNex® Size-Selective Purification System (Promega). Sequencing was conducted using the Illumina MiSeq platform (2 × 300 bp paired-end) at CJ Bioscience, Inc. (Seoul, Republic of Korea). Negative controls (extraction blanks and PCR blanks) and positive controls (mock community) were not included in this study. All laboratory steps were performed under sterile, contamination-controlled conditions following standardized CJ Bioscience workflows.

### Sequence processing and taxonomic assignment

Initial quality filtering was performed with Cutadapt (v3.2) to remove adapter and primer sequences [12]. Reads were truncated at the first base with a quality score ≤ Q20 using the filterAndTrim function in the DADA2 R package (v1.20.0) [13]. Forward and reverse reads were merged using mergePairs, and chimeric sequences were removed using removeBimeraDenovo. Amplicon sequence variant (ASV) tables were inferred using the DADA2 plugin in QIIME2 (v2021.11) [14], which performs denoising, error correction, and chimera removal while automatically excluding spurious singleton reads. Accordingly, singleton ASVs were automatically excluded, and no additional singleton filtering was required. No additional decontamination steps or manual removal of ASVs were applied beyond the standard DADA2 pipeline. Per-sample sequencing statistics at each step of the QIIME2–DADA2 pipeline are summarized in Supplementary Table S1. The mean raw sequencing depth was approximately 82,500 paired-end reads per sample, and after quality filtering and chimera removal, the median final read depth was 38,262 reads per sample (IQR 16,304; range 17,173 – 81,417). No samples were excluded due to low sequencing depth, and all fecal samples were included in downstream analyses. Taxonomic assignment was performed using the QIIME2 plugin q2-feature-classifier classify-consensus-vsearch against the EzBioCloud 16S rRNA gene database (v4.1, November 18, 2022) [15]. Taxa were identified with ≥ 97% sequence identity and ≥ 80% coverage. Species-level identifications were considered putative because the V3–V4 16S region does not always provide sufficient resolution to distinguish closely related species.

### Microbiome analysis

All downstream microbiome analyses were performed using R software (v4.2.0). The “phyloseq” R package (v1.50.0) was used for data preprocessing, taxonomic aggregation, and diversity analysis [16]. Rarefaction curves (sampling depth vs. observed ASVs) were generated using the *vegan:rarecurve()* function to assess sequencing depth across samples. As shown in Supplementary Figure S1, all samples reached a clear plateau, indicating sufficient sequencing coverage. Because all samples exceeded the saturation point (final median depth ≈38,000 reads/sample), no rarefaction-based subsampling cutoff was applied. Instead, diversity and differential abundance analyses were performed using relative abundance normalization to the total reads per sample, minimizing bias from uneven sequencing depth. Alpha diversity indices, including Chao1 [17], Shannon, and Simpson [18], were calculated using the QIIME2 DADA2 plugin and compared between groups using the Wilcoxon rank-sum test [19]. Beta diversity was assessed by calculating Bray–Curtis dissimilarity and visualized via Principal Coordinate Analysis (PCoA). Group differences in community structure were tested by PERMANOVA with 999 permutations using the “vegan” R package (v2.6–10) [20, 21].

### Biomarker discovery

To identify differentially abundant microbial taxa between SLE and HC groups, Linear Discriminant Analysis Effect Size (LEfSe) was performed using the “microbiomeMarker” R package (v1.13.2) [22, 23]. Taxa with a Kruskal–Wallis test *p* value ≤ 0.05 and Wilcoxon test *p* value ≤ 0.05 were considered significant. Linear Discriminant Analysis (LDA) score thresholds were set to ≥ 2.0 at the genus level and ≥ 1.0 at the species level.

### Clinical correlation analysis

Associations between relative abundances of key microbial taxa and clinical parameters (e.g., disease activity index, presence of LN) were assessed using Spearman’s rank correlation [24]. Correlation matrices were visualized as heatmaps using custom R scripts with the “ggplot2” package (v3.3.5) [25]. Significance levels were indicated as *p* < 0.05 (*), *p* < 0.01 (**), and *p* < 0.001 (***).

### Batch handling and confounders

All fecal samples were extracted using the same Maxwell RSC PureFood GMO and Authentication Kit (Promega) and processed in a randomized and blinded manner to ensure that sample order and sequencing batch were unrelated to disease/control status. Given that all SLE patients were on treatment and dietary control was not feasible in this real-world clinical cohort, multivariable regression for medication or diet was not performed. However, additional correlation analyses incorporating key clinical covariates were performed and are presented in Supplementary Figure S2.

### Functional prediction of microbial metabolism

Functional profiles of the gut microbiota were inferred using the PICRUSt2 pipeline (v2.4.1) [26], implemented in QIIME2 (v2021.11) and CJ Bioscience’s EzBioCloud 16S-based MTP analysis platform. The accuracy of metagenomic inference was evaluated by calculating the Nearest Sequenced Taxon Index (NSTI) for each sample. Across all samples, the mean NSTI was 0.021 ± 0.013 (median 0.017, IQR 0.010, range [min–max] 0.005–0.094), indicating that most ASVs were closely related to sequenced reference genomes and that the predicted functional profiles were highly reliable. Predicted functions were annotated using the KEGG Orthology (KO) and Enzyme Commission (EC) classification systems [27]. Relative abundances of predicted pathways and enzymatic functions were compared between the SLE and HC groups. Functional differences were visualized via bar plots. Statistical comparisons were conducted using Welch’s t-test followed by Benjamini–Hochberg false discovery rate (FDR) correction, and pathways with adjusted *p* values ≤ 0.05 were considered significant.

## Results

### Study population characteristics

A total of 157 Korean patients with SLE and 50 HC were enrolled. The clinical and demographic characteristics of the participants are summarized in Table 1. The median age and body mass index were comparable between the two groups, and most participants in both groups were female. Among the SLE cohort, 64 patients (40.8%) had a history of LN, and 42 patients (26.8%) had high disease activity (SLEDAI ≥ 7). Most SLE patients were being treated with hydroxychloroquine (89.2%) and glucocorticoids (85.4%).

**Table 1:** Demographic and clinical information of patients with systemic lupus erythematosus (SLE) and healthy controls (HC)

	Total SLE (*n* = 157)	HC (*n* = 50)
Age, years	41 (31–49)	39 (29–44)
Sex, female	141 (89.8)	45 (90.0)
Body mass index, kg/m^2^	21.7 (19.5–23.7)	21.7 (19.9–24.4)
Disease duration, months	112 (46–222)	
Lupus nephritis	64 (40.8)	
SLEDAI	4 (2–7)	
High SLEDAI (SLEDAI ≥ 7)	42 (26.8)	
SLICC/ACR damage index	1 (0–1)	
Treatment		
Glucocorticoid	134 (85.4)	
Dose of glucocorticoid*, mg/day	2.5 (2.5–5.0)	
Hydroxychloroquine	140 (89.2)	
Belimumab	19 (12.1)	
Gastrointestinal medication	95 (60.5)	
Serum C3, mg/dL	81 (68–93)	
Serum C4, mg/dL	16.9 (9.7–21.6)	
Serum IgG, mg/dL	1431 (1191–1711)	
Anti-DNA antibody, IU/mL	21.30 (5.14–71.13)	

Data are shown as *n* (%) or median (interquartile range). *Glucocorticoid dose is presented as prednisolone-equivalent (mg/day). SLEDAI: Systemic Lupus Erythematosus Disease Activity Index, SLICC: Systemic Lupus International Collaborating Clinics, ACR: American College of Rheumatology

### Gut microbial diversity is reduced in SLE patients

**Fig. 1:**
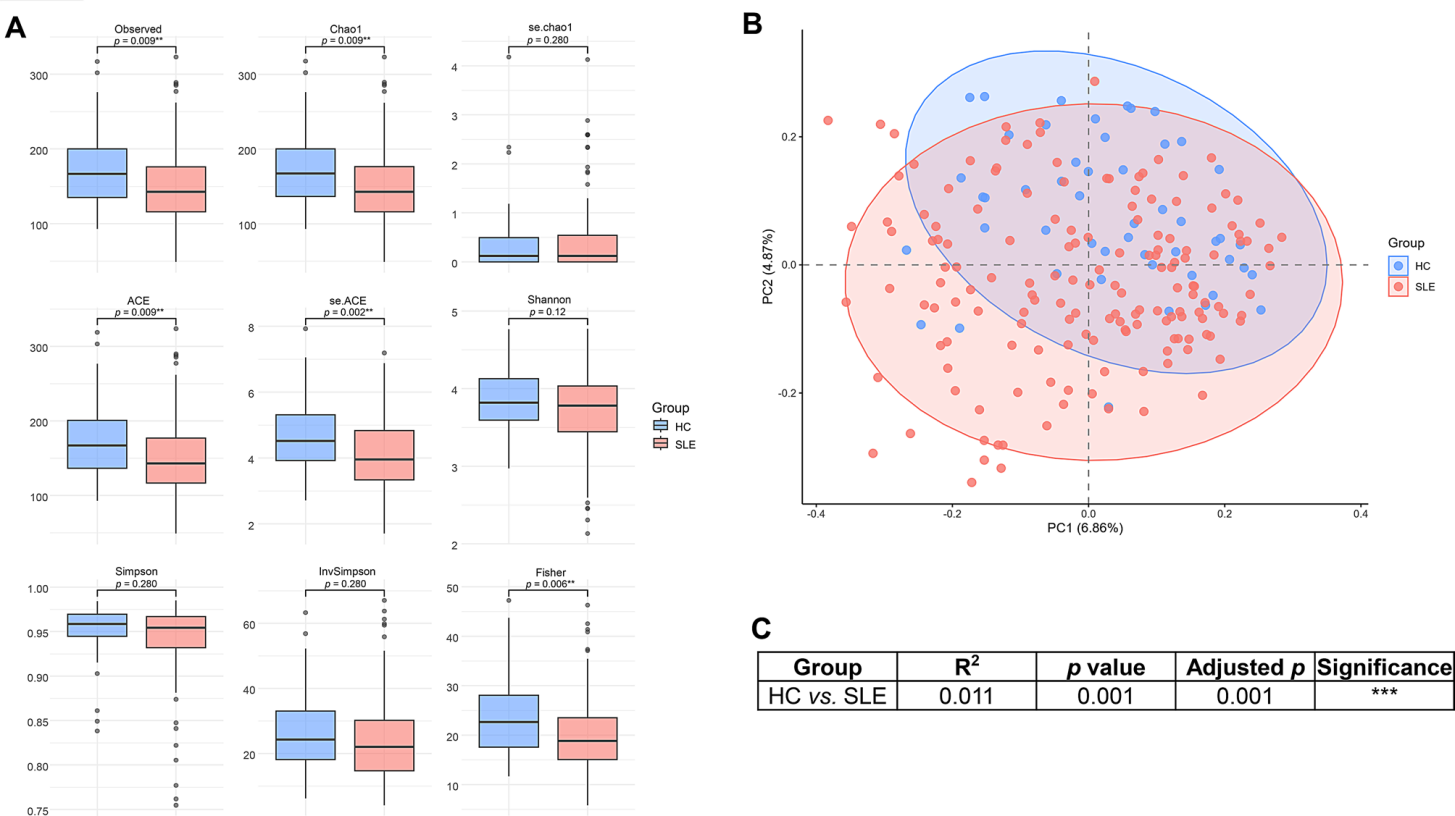
Comparison of gut microbial diversity between systemic lupus erythematosus (SLE) patients and healthy controls (HC). (**A**) Boxplots of alpha diversity indices including observed ASVs, Chao1, ace, Shannon, Simpson, inverse Simpson, and Fisher index. Statistical significance was determined using the Wilcoxon rank-sum test. Boxes represent interquartile ranges (IQRs), horizontal lines indicate medians, and whiskers represent 1.5 × IQR; outliers are shown as dots. (**B**) Principal coordinate analysis (PCoA) plot based on Bray–Curtis dissimilarity demonstrates distinct clustering of gut microbial communities in SLE and HC groups. Statistical significance was assessed by PERMANOVA using 999 permutations. Each dot represents an individual sample, and ellipses indicate 95% confidence intervals for each group. (**C**) Table summarizing PERMANOVA results comparing overall beta diversity between HC and SLE groups. Significance levels are indicated as follows; ns: not significant, *: *p* < 0.05, **: *p* < 0.01, ***: *p* < 0.001

To evaluate the gut microbial diversity, we analyzed alpha and beta diversity between SLE patients and HC. Alpha diversity, which reflects the richness of microbial communities, was significantly lower in the SLE group across multiple indices, including Chao1 (*p* = 0.009), ACE (*p* = 0.009) [28], and Fisher (*p* = 0.006) [29] (Fig. 1A). This suggests an overall reduction in microbial diversity in SLE. However, the Shannon and Simpson indices did not show statistically significant differences between the two groups, suggesting that the evenness of the microbial communities remained relatively unaffected. Beta diversity assessed by Bray–Curtis distance and visualized using PCoA revealed distinct clustering of the SLE and HC groups (Fig. 1B), indicating altered overall microbial composition. The differences were statistically significant as confirmed by PERMANOVA (Fig. 1C, R^2^ = 0.011, *p* = 0.001, adjusted *p* = 0.001). These results suggest that both richness and community structure of the gut microbiota are markedly altered in SLE patients.

### Altered microbial composition in SLE patients

**Fig. 2:**
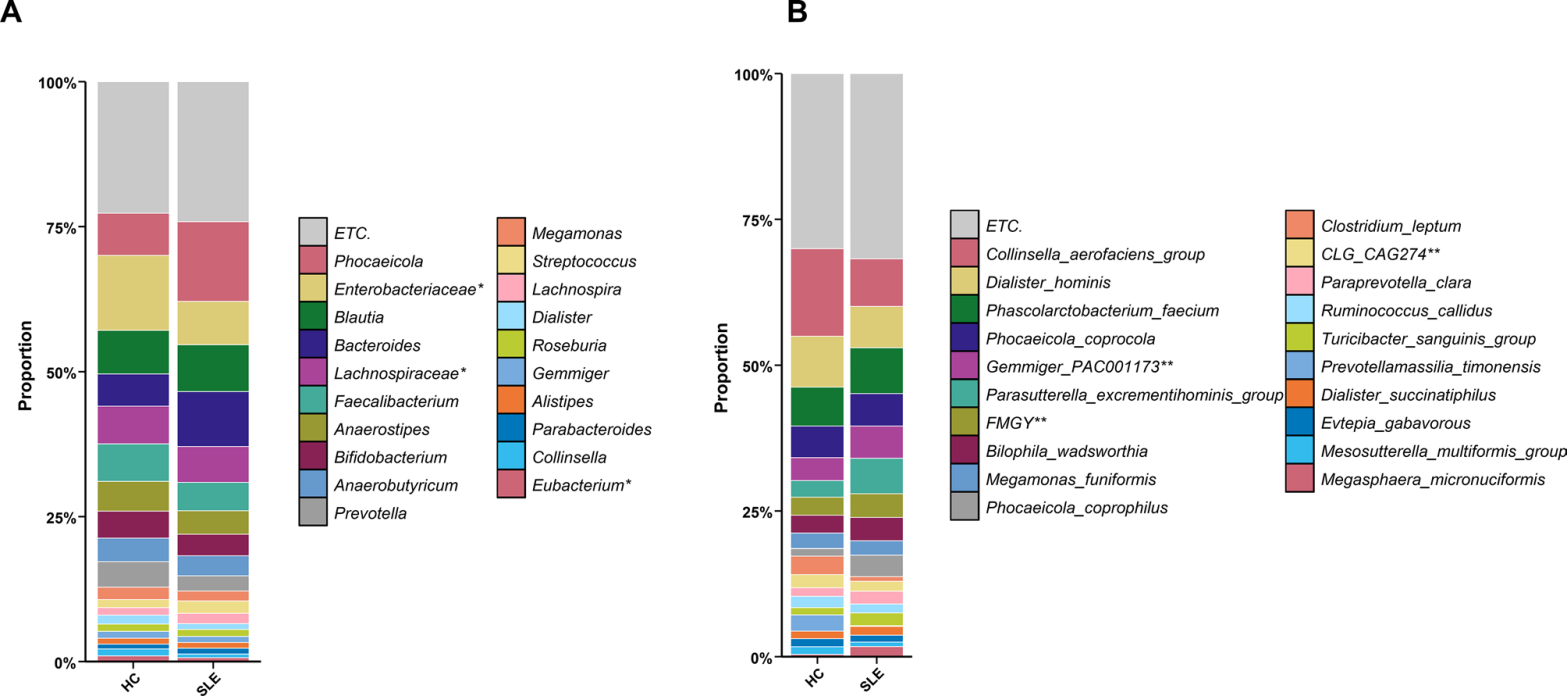
Taxonomic composition of the gut microbiota in HC and SLE patients. (**A**) Stacked bar plots showing the average relative abundance of the top 20 bacterial genera in the HC and SLE groups. (**B**) Stacked bar plots displaying the average relative abundance of the top 20 bacterial species by group. Taxa with lower mean abundance were grouped into the “ETC.” category to improve visualization. Each color denotes a distinct genus (**A**) or species (**B**), and bars represent the mean microbial composition within each group. Asterisks (*) indicate unclassified genera. Double asterisks (**) denote provisional species-level assignments based on marker gene clustering

**Fig. 3:**
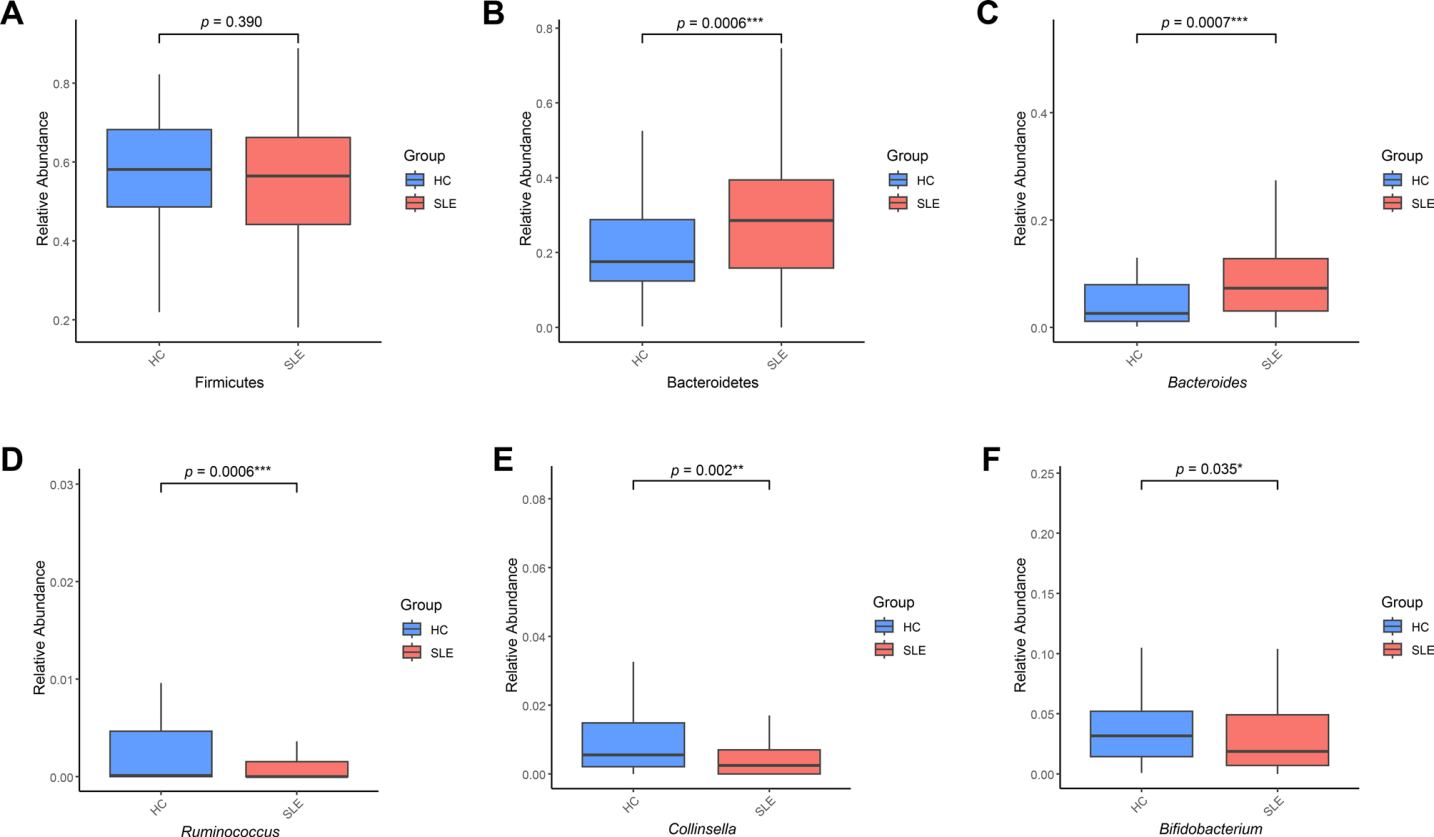
Relative abundance of key microbial taxa in SLE and HC groups. This figure illustrates the relative abundance of selected gut microbial taxa in HC (*n* = 50, blue) and SLE (*n* = 157, red) groups using boxplots. Each boxplot displays the median, interquartile range (IQR), and outliers. Comparisons were made using the Wilcoxon rank sum test. (**A**) Firmicutes (phylum level), (**B**) Bacteroidetes (phylum level), (**C**) *Bacteroides* (genus level), (**D**) *Ruminococcus* (genus level), (**E**) *Collinsella* (genus level), (**F**) *Bifidobacterium* (genus level). Significance levels are indicated as follows; ns: not significant, *: *p* < 0.05, **: *p* < 0.01, ***: *p* < 0.001

Next, we examined the composition of the gut microbiota in both groups. Stacked bar plots showed marked differences in the relative abundance of dominant bacterial genera and species between SLE and HC (Fig. 2A–B). Among the top 20 genera, shifts in the abundance of *Bacteroides*, *Faecalibacterium*, *Collinsella*, and *Bifidobacterium* were particularly notable (Fig. 2A). At the species level, within the top 20 most abundant taxa, *Collinsella aerofaciens* and *Clostridium leptum* were decreased in the SLE group compared to HC, whereas *Phocaeicola coprophilus* was increased (Fig. 2B). Next, we compared the relative abundance of specific bacterial taxa previously reported to be associated with SLE between the SLE and HC groups. At the phylum level, the SLE group showed a slightly lower proportion of Firmicutes compared to controls, although the difference was not statistically significant (Fig. 3A). In contrast, the proportion of Bacteroidetes was significantly higher in the SLE group (Fig. 3B, *p* < 0.001). At the genus level, *Bacteroides* was enriched in SLE (*p* < 0.001), while *Ruminococcus* (*p* < 0.001), *Collinsella* (*p* = 0.002) and *Bifidobacterium* (*p* = 0.035) were significantly decreased (Fig. 3C–F). These findings indicate SLE-associated dysbiosis characterized by the loss of known beneficial microbes and enrichment of pathobionts.

### LEfSe identifies distinct microbial taxa enriched in SLE

**Fig. 4:**
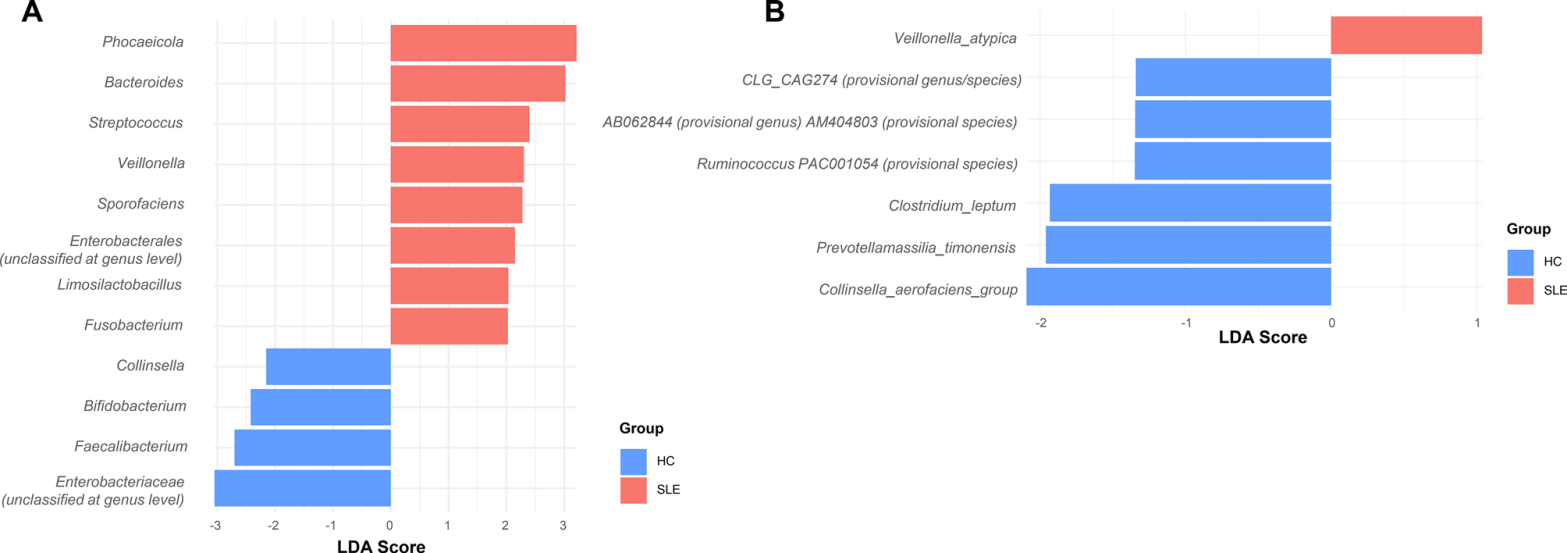
Discriminative microbial taxa between SLE and HC groups based on LEfSe analysis. This figure presents linear discriminant analysis (LDA) scores of microbial features that significantly differentiate the SLE (*n* = 157, red) and HC (*n* = 50, blue) groups. (**A**) Genus-level biomarkers: only genera with lda scores ≥ 2.0 are shown. (**B**) Species-level biomarkers: species with lda scores ≥ 1.0 are presented. Taxa selection was based on Kruskal–Wallis followed by Wilcoxon rank-sum tests (*p* ≤ 0.05). Color bars represent the group in which the taxon is relatively enriched: red for SLE and blue for HC

To identify specific microbial taxa that distinguish SLE patients from HC, we performed LEfSe analysis. All features with an LDA score ≥ 2 (genus level) or ≥ 1 (species level) and *p* ≤ 0.05 were included. At the genus level, SLE patients showed significant enrichment of *Phocaeicola*, *Bacteroides*, *Streptococcus* and *Veillonella*, while *Collinsella*, *Faecalibacterium*, and *Bifidobacterium* were enriched in the HC group (Fig. 4A). At the species level, *Veillonella atypica* was enriched in the SLE group, whereas *Collinsella aerofaciens* group, *Clostridium leptum*, and other taxa such as *Prevotellamassilia timonensis* were more abundant in HC (Fig. 4B). To further validate these findings, we calculated adjusted *p* values using the Benjamini–Hochberg FDR correction and corresponding log₂ fold changes for all taxa identified as potential biomarkers. As shown in Supplementary Table S2 and S3, most of the taxa enriched in SLE or HC remained significant after FDR adjustment, supporting the robustness of the identified microbial signatures. These taxa may represent microbial signatures associated with SLE-related gut dysbiosis. To explore potential associations between clinical factors and gut microbial biomarkers, correlation analyses were performed using Spearman’s rank correlation. The resulting heatmap (Supplementary Figure S2) illustrates the relationships between key SLE-related parameters (e.g., SLEDAI, complement, anti-dsDNA antibody levels) and the relative abundance of major microbial taxa.

### Subgroup analyses: nephritis status and disease activity within SLE patients

**Fig. 5:**
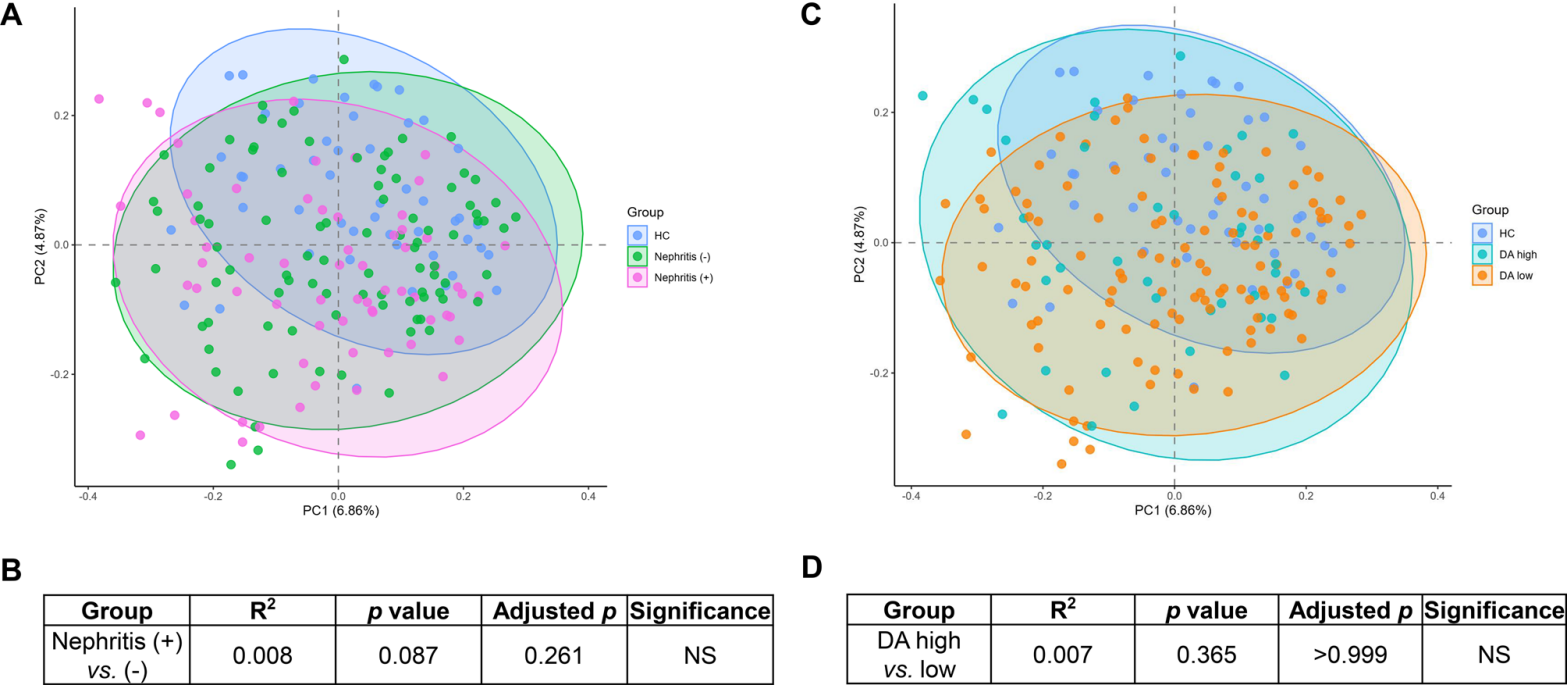
Gut microbial beta diversity in sle patients stratified by nephritis status and disease activity. (**A**) PCoA plot based on Bray–Curtis distances comparing beta diversity of the gut microbiota among healthy controls (HC, *n* = 50, blue), SLE patients without lupus nephritis (LN−, *n* = 93, green), and SLE patients with lupus nephritis (LN+, *n* = 64, pink). Statistical significance was assessed by PERMANOVA using 999 permutations. Each point represents an individual sample, and ellipses indicate the 95% confidence interval for each group. (**B**) Statistical comparison of beta diversity between LN+ and LN− groups using PERMANOVA. The table presents unadjusted and multiple comparison-adjusted *p* values. (**C**) PCoA plot based on Bray–Curtis distances comparing HC (blue), SLE patients with high disease activity (DA high, *n* = 42, turquoise; SLEDAI ≥ 7), and low disease activity (DA low, *n* = 115, orange; SLEDAI < 7). Statistical significance was assessed by PERMANOVA using 999 permutations. Ellipses represent 95% confidence intervals. (**D**) PERMANOVA results comparing DA high and DA low groups. Significance levels are indicated as follows; ns: not significant

We investigated whether the presence of LN or differences in disease activity were associated with distinct gut microbial patterns within the SLE group. Beta diversity analysis using Bray–Curtis distance and PCoA showed no clear separation between nephritis-positive and nephritis-negative SLE patients (Fig. 5A), nor between high and low disease activity (DA) subgroups (Fig. 5C). PERMANOVA results confirmed that there were no statistically significant differences in community composition between nephritis-positive and nephritis-negative patients (R^2^ = 0.008, *p* = 0.087, adjusted *p* = 0.261, Fig. 5B), or between high and low DA groups (R^2^ = 0.007, *p* = 0.365, adjusted *p* > 0.999, Fig. 5D). To further explore this, we analyzed alpha diversity within these SLE subgroups. As shown in Supplementary Figure S3, alpha diversity indices (Chao1, Shannon, ACE) showed no significant difference between the two SLE subgroups based on the presence of nephritis. Similarly, Supplementary Figure S4 shows that while both high and low DA SLE groups had reduced diversity compared to HC in some indices, the difference between DA-high and DA-low subgroups was not statistically significant. These results should be interpreted with caution, as the limited subgroup sizes and the inherent variability of microbiome data may have reduced statistical power to detect subtle differences. Within these constraints, our findings suggest that gut microbial alterations observed in this cohort represent a shared dysbiosis pattern across SLE patients rather than clearly distinct signatures driven by nephritis or disease activity.

### Predicted microbial metabolic functions show minimal differences between SLE and controls

We performed PICRUSt2-based functional prediction analysis to investigate whether gut microbial alterations in SLE are accompanied by shifts in microbial metabolic potential. KO and EC profiles were inferred from 16S rRNA gene data. The NSTI scores across all samples were low (mean 0.021 ± 0.013), suggesting high accuracy of the PICRUSt2-based functional inference. LEfSe analysis initially identified several EC- and KO-level features, including pathways related to carbohydrate metabolism and membrane transport, that appeared more enriched in SLE, whereas a few enzymatic functions were relatively enriched in HC (Supplementary Figure S5). However, these differences did not remain statistically significant after FDR correction. Thus, although some nominal variations were observed, the predicted functional capacity of the microbiome was largely conserved between SLE patients and HC.

## Discussion

In this study, we characterized the gut microbiome of Korean patients with SLE compared with HC. SLE patients exhibited distinct gut microbial community structures, accompanied by compositional shifts such as an increased abundance of *Veillonella atypica* and a decrease in *Collinsella aerofaciens*. Functional prediction using PICRUSt2 suggested potential alterations in metabolic pathways. However, no features remained statistically significant after FDR correction. Moreover, microbial profiles did not show clear separation when stratified by LN status or overall disease activity, suggesting that the gut microbial perturbations observed in SLE reflect disease-related systemic immune dysregulation rather than organ-specific or activity-dependent changes. Collectively, these findings are consistent with previous studies demonstrating gut dysbiosis in SLE and reinforce the concept that microbial imbalance may play a role in the chronic immune activation underlying this disease.

We observed significantly reduced alpha diversity in the gut microbiota of SLE patients, particularly in richness-based indices such as Chao1, ACE, and Fisher. This aligns with previous reports indicating lower microbial richness in SLE populations across various ethnic groups, including Chinese, French, and American cohorts [3, 4, 30]. The relatively preserved Shannon and Simpson indices suggest that while the number of species is reduced, the evenness of remaining taxa is maintained. This pattern implies a narrowing of microbial repertoire without dominance of any single species. Reduced diversity may compromise colonization resistance, immune education, and mucosal tolerance, thereby increasing the susceptibility to systemic autoimmunity [31]. It has been shown that microbial diversity influences SCFA production, which promotes regulatory T cells and gut barrier integrity [32]. Therefore, diversity loss may exacerbate systemic inflammation in SLE via impaired immune modulation.

Taxonomic analysis revealed a notable decrease in *Collinsella*, *Ruminococcus*, and *Bifidobacterium* in SLE patients, alongside enrichment of *Bacteroides*, *Streptococcus*, and *Veillonella*. These observations partially overlap with findings from other cohorts. For instance, *Collinsella *has been previously reported to be altered in rheumatoid arthritis, where its abundance associated with inflammatory activity [33, 34]. Notably, our study is the first to demonstrate a depletion of *Collinsella aerofaciens* in patients with SLE. *Bifidobacterium* spp., known for their mucin-degrading and gut barrier-enhancing roles, were also reduced in our cohort, consistent with the hypothesis that loss of barrier-protective microbes facilitates microbial translocation and immune activation [35]. The enrichment of *Veillonella atypica*, a known lactic acid–utilizing anaerobe, is particularly interesting. *Veillonella* has been implicated in promoting inflammatory cytokine production and has been enriched in patients with inflammatory diseases in prior metagenomic studies [36, 37]. Because 16S rRNA V3–V4 amplicon sequencing provides limited resolution for closely related taxa, these species-level annotations (e.g., *Veillonella atypica*, *Collinsella aerofaciens*) should be regarded as putative assignments. Future studies employing shotgun metagenomics or targeted qPCR will be necessary to validate the species-level identities of these key microbial signatures. Similarly, *Streptococcus* species, particularly oral pathobionts, have been found in elevated abundance in SLE patients and may contribute to systemic inflammation through translocation or mimicry [30, 38]. Importantly, although the Firmicutes/Bacteroidetes ratio was altered in our study (with increased Bacteroidetes), the difference was not statistically robust at the phylum level. This suggests that compositional changes at the genus and species levels may be more informative in SLE than phylum-level metrics, especially given the functional redundancy within phyla.

LEfSe analysis identified *Phocaeicola*, *Bacteroides*, and *Veillonella* as enriched in SLE patients, while *Collinsella*, *Faecalibacterium*, and *Bifidobacterium* were more abundant in HC. These taxa may serve as candidate microbial biomarkers for SLE-associated dysbiosis. Notably, *Faecalibacterium prausnitzii*, a SCFA-producing species with known anti-inflammatory effects, was significantly depleted in the SLE group [39]. This pattern has been consistently observed across other autoimmune diseases, including inflammatory bowel disease and type 1 diabetes [40]. It remains uncertain whether these taxa contribute to disease pathogenesis or merely reflect an altered immune environment. However, Chen et al. demonstrated that certain SLE-enriched bacteria can produce immunogenic peptides that mimic autoantigens and activate peripheral immune responses, thereby directly contributing to disease progression [3]. Whether similar mechanisms are operative for *Veillonella* or *Phocaeicola* in Korean patients warrants further investigation.

Contrary to several previous reports, we found no significant difference in microbial composition between SLE patients with and without LN, or between those with high versus low disease activity. This suggests that gut dysbiosis in SLE may be a general disease feature rather than one that reflects specific organ involvement or flare status. Our findings differ from those of Azzouz et al., who reported enrichment of *Ruminococcus gnavus* in SLE patients with active nephritis [41]. One possible explanation for this discrepancy lies in ethnic and geographic differences, which shape microbiota via host genetics, dietary patterns, and environmental exposures [42]. In addition, most patients in our cohort were on stable immunosuppressive treatment, which may have attenuated inter-group microbial variation. Moreover, a study by Guo et al. found distinct microbiota patterns in treatment-naïve Chinese patients compared to those receiving therapy [43]. Therefore, immunosuppressants, particularly glucocorticoids and hydroxychloroquine, may modulate gut microbial profiles in a way that masks associations with disease severity or nephritis status. This underscores the importance of treatment stratification and longitudinal study design in future microbiome-SLE research.

PICRUSt2-based functional prediction revealed minimal differences in predicted metabolic pathways between SLE and HC. Although minor shifts in carbohydrate metabolism and membrane transport were noted, none reached statistical significance after FDR correction. This suggests that despite taxonomic dysbiosis, the microbial community may retain a degree of functional redundancy, which is a concept supported by previous reports in other chronic inflammatory diseases [44, 45]. Although the low NSTI values in our dataset (mean 0.021 ± 0.013) further support the reliability of the predicted functional profiles, 16S-based functional prediction has inherent limitations, including its inability to capture strain-level variation or microbial gene expression. Therefore, future studies using metagenomic shotgun sequencing or metatranscriptomics are warranted to better understand how microbial functionality contributes to SLE immunopathogenesis.

This study has several limitations. First, its cross-sectional design precludes causal inferences about the role of gut dysbiosis in SLE. Although regression models adjusting for confounders could provide additional robustness, most SLE patients were on similar treatment regimens, limiting variability for model-based adjustment. Instead, HC were matched for age, sex, and BMI to minimize baseline differences. Thus, non-parametric tests were used for group comparisons of diversity indices. Second, although we collected limited dietary information (smoking, coffee, and alcohol consumption), these data were descriptive and not standardized for quantitative analysis; thus, dietary factors were not included as covariates. In addition, most SLE patients were receiving glucocorticoids, hydroxychloroquine, gastrointestinal medications, or belimumab at stable doses, which may influence gut microbial composition. While these factors were not controlled for statistically, exploratory correlation analyses between major microbial taxa and clinical or medication variables were performed and are shown in Supplementary Figure S2. Future studies using longitudinal sampling and controlled dietary conditions will be necessary to better delineate medication- and diet-related effects on gut microbiota in SLE. Third, functional predictions were based on 16S data, which lack resolution compared to whole metagenome sequencing. Nevertheless, our study provides a comprehensive snapshot of gut microbiota in a relatively large, well-characterized Korean SLE cohort, offering a valuable reference point for future multi-ethnic or longitudinal microbiome investigations.

Our study confirms that gut dysbiosis is a consistent feature of SLE, even in a Korean population, but highlights the complexity and variability of its taxonomic and functional patterns. Given the absence of strong associations with disease activity or nephritis status, gut microbiota may be more useful as a diagnostic marker rather than a monitoring tool. However, it is also possible that gut microbial alterations precede clinical flare or organ involvement. This hypothesis requires longitudinal studies for validation. Furthermore, interventions aimed at modulating the gut microbiome, such as probiotics, prebiotics, dietary modification, or fecal microbiota transplantation, are being actively explored in autoimmune diseases. Whether such strategies can reduce disease flares or improve therapeutic outcomes in SLE remains an exciting avenue for future research.

In summary, our study demonstrates distinct gut microbiome alterations in Korean SLE patients, characterized by reduced microbial diversity and compositional shifts favoring proinflammatory taxa. These changes were not significantly associated with disease activity or renal involvement, suggesting a global dysbiosis pattern in SLE. Our findings add to the growing body of evidence supporting the gut–immune axis in lupus pathogenesis and highlight the potential of microbiome profiling as a diagnostic and therapeutic avenue.

## Electronic supplementary material

Below is the link to the electronic supplementary material.

Supplementary Material 1

## Data Availability

The datasets supporting the conclusions of this article have been deposited in the Korean BioData Station (K-BDS) repository under accession number KAP241782. Due to funding conditions the data are under restricted access but will become publicly accessible on 31 December 2027. Requests for access prior to that date may be addressed to the corresponding author.
