# Supplementary material for: Distinct gut microbiome profiles in Korean systemic lupus erythematosus patients
Source: J Transl Med. 2025 Dec 23;23:1426. doi: 10.1186/s12967-025-07438-7 (PMC12739845; doi:10.1186/s12967-025-07438-7)
Supplement: Supplementary file 1 — Supplementary Material 1 [file 12967_2025_7438_MOESM1_ESM.docx]

**Supplementary Materials**

**Table S1. Summary of sequencing read counts per sample at each preprocessing step.**

| **Step** | **Median** | **IQR** | **Range (Min–Max)** |
| --- | --- | --- | --- |
| Raw reads | 46,049 | 19,809 | 20,151 – 115,942 |
| After CutAdapt (primer/adaptor removal) | 46,049 | 19,809 | 20,151 – 115,942 |
| After quality filtering | 45,946 | 20,197 | 20,048 – 115,851 |
| After denoising (forward) | 44,433 | 19,845 | 19,078 – 110,996 |
| After denoising (reverse) | 43,212 | 19,164 | 18,603 – 108,114 |
| After merging | 40,272 | 17,536 | 17,256 – 95,344 |
| After chimera removal (final reads) | 38,262 | 16,304 | 17,173 – 81,417 |

Values represent total read counts per sample before and after successive filtering steps. Median, interquartile range (IQR), and range are shown.


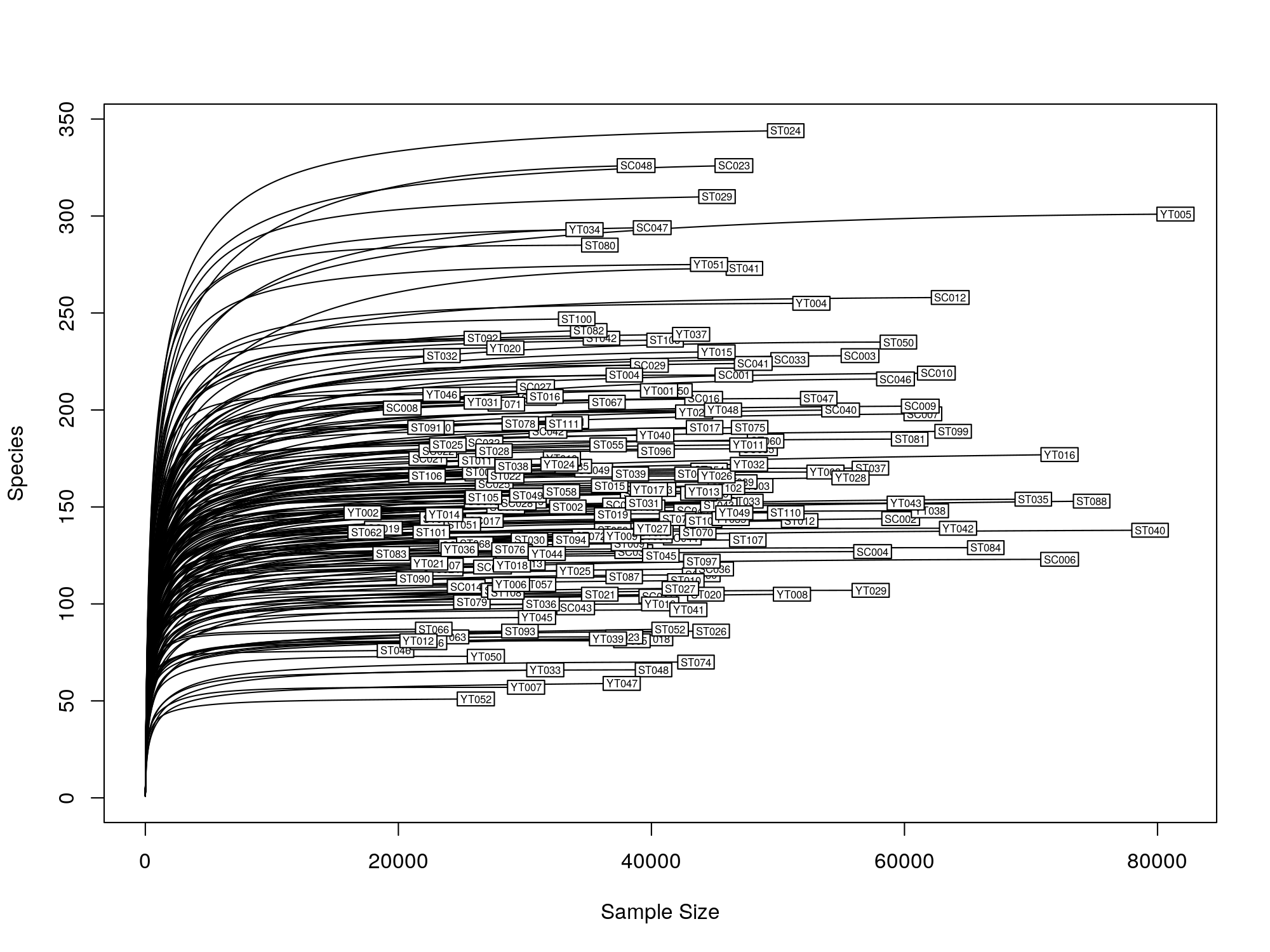


**Figure S1.** Rarefaction curves of observed amplicon sequence variants (ASVs) for all fecal samples.

**Table S2.** Differential abundance analysis of gut microbial taxa with false discovery rate (FDR)-corrected *p* values and log₂ fold changes at genus level.

| **Feature** | **Enriched Group** | **LDA Score** | ***p* value** | **FDR** |
| --- | --- | --- | --- | --- |
| *Enterobacteriaceae_g* | HC | 3.0605 | 0.0002 | 0.0002 |
| *Faecalibacterium* | HC | 2.7317 | 0.0312 | 0.0312 |
| *Bifidobacterium* | HC | 2.2078 | 0.0277 | 0.0277 |
| *Collinsella* | HC | 2.0865 | 0.0020 | 0.0020 |
| *Phocaeicola* | SLE | 3.2210 | 0.0036 | 0.0036 |
| *Bacteroides* | SLE | 2.9978 | 0.0008 | 0.0008 |
| *Streptococcus* | SLE | 2.4769 | 0.0010 | 0.0010 |
| *Veillonella* | SLE | 2.4399 | 0.0000 | 0.0000 |
| *Sporofaciens* | SLE | 2.3539 | 0.0193 | 0.0193 |
| *Enterobacterales_f_g* | SLE | 2.1099 | 0.0030 | 0.0030 |
| *Fusobacterium* | SLE | 2.0723 | 0.0266 | 0.0266 |
| *Limosilactobacillus* | SLE | 2.0198 | 0.0001 | 0.0001 |

Taxa with FDR < 0.05 were considered significantly different between SLE patients and HC.

**Table S3.** Differential abundance analysis of gut microbial taxa with false discovery rate (FDR)-corrected *p* values and log₂ fold changes at species level.

| **Feature** | **Enriched Group** | **LDA Score** | ***p* value** | **FDR** |
| --- | --- | --- | --- | --- |
| *Collinsella_aerofaciens_group* | HC | 2.1024 | 0.0018 | 0.0018 |
| *Prevotellamassilia_timonensis* | HC | 1.8727 | 0.0005 | 0.0005 |
| *Clostridium_leptum* | HC | 1.8684 | 0.0097 | 0.0097 |
| *CLG_CAG274_g_CLG_CAG274_s* | HC | 1.3832 | 0.0002 | 0.0002 |
| *Ruminococcus_PAC001054_s* | HC | 1.3761 | 0.0054 | 0.0054 |
| *AB062844_g_AM404803_s* | HC | 1.3065 | 0.0187 | 0.0187 |
| *Veillonella_atypica* | SLE | 1.0350 | 0.0082 | 0.0082 |

Taxa with FDR < 0.05 were considered significantly different between SLE patients and HC.


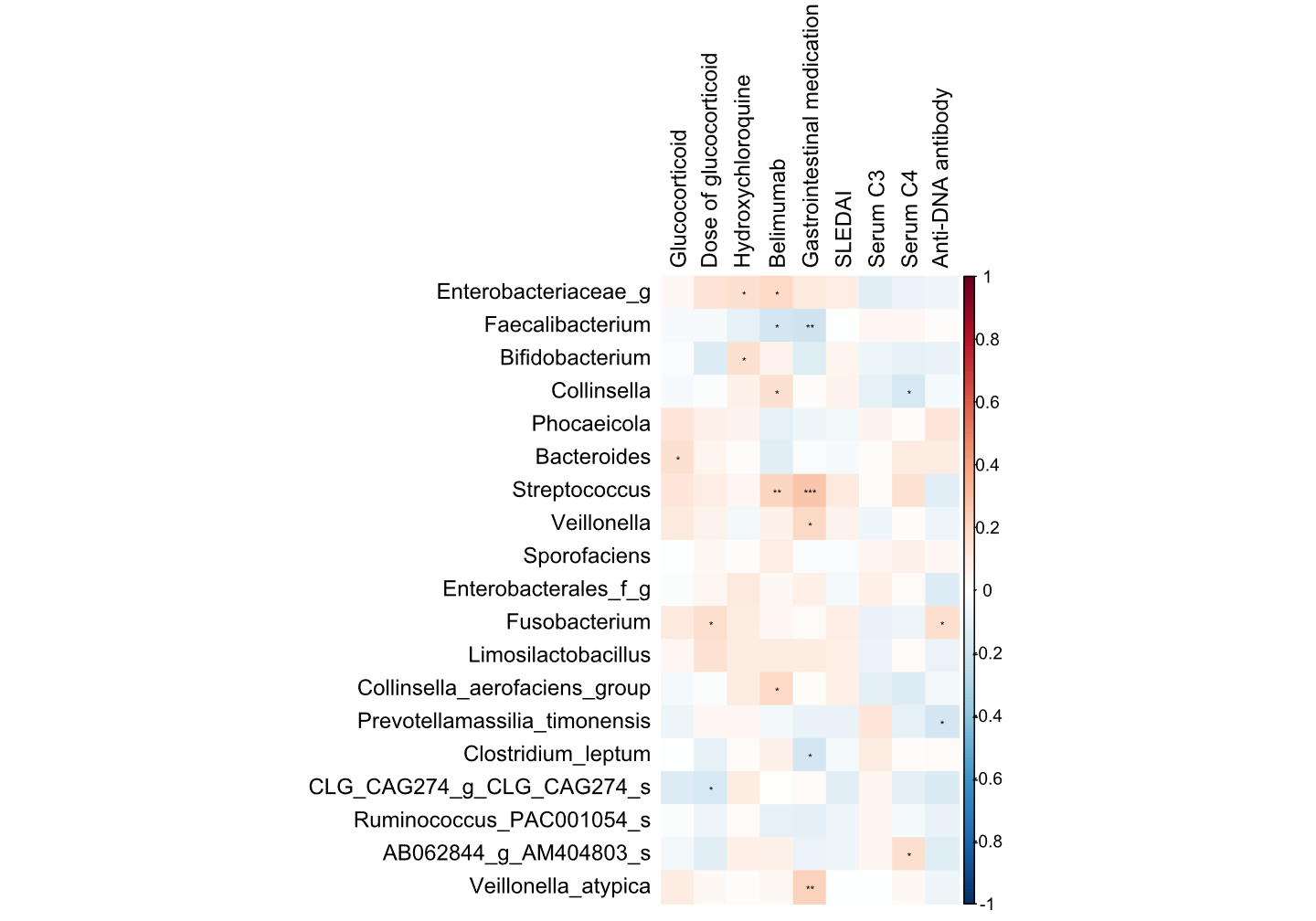


**Figure S2**. Correlation between major microbial taxa and clinical/medication variables in SLE patients. The heatmap shows Spearman correlation coefficients between relative abundance of representative bacterial taxa and clinical variables including glucocorticoid dose, hydroxychloroquine and belimumab use, gastrointestinal medication, SLEDAI, serum complement (C3, C4), and anti-dsDNA antibody levels. *: *p* < 0.05, **: *p* < 0.01, ***: *p* < 0.001.

**
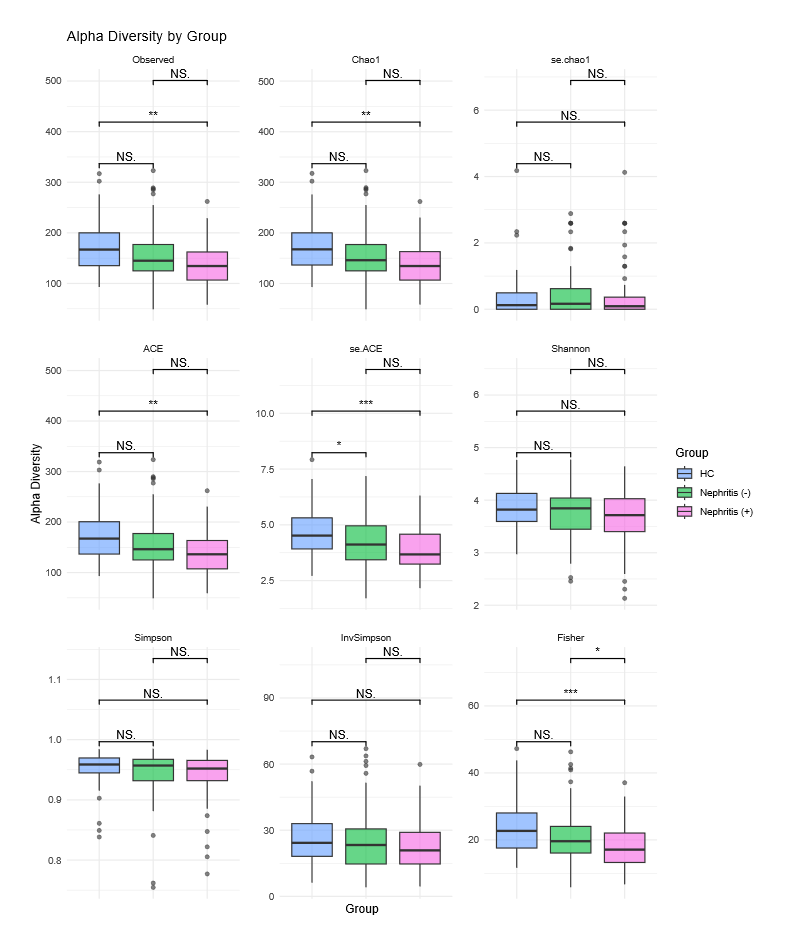
**

**Figure S3.** Alpha diversity of gut microbiota in lupus nephritis (LN) subgroups and healthy controls. Boxplots illustrate the alpha diversity metrics across healthy controls (HC, *n* = 50, blue), SLE patients without nephritis (LN−, *n* = 93, green), and SLE patients with nephritis (LN+, *n* = 64, pink). Nine diversity indices are presented, including Observed species, Chao1, ACE, Shannon, Simpson, inverse Simpson, and Fisher's alpha, each capturing different aspects of species richness and evenness. Boxes indicate the median and interquartile range (IQR), with whiskers showing 1.5 × IQR and dots representing outliers. Statistical significance was evaluated using the Wilcoxon rank-sum test for pairwise comparisons. Significance levels are indicated as follows; NS: not significant, *: *p* < 0.05, **: *p* < 0.01, ***: *p* < 0.001.


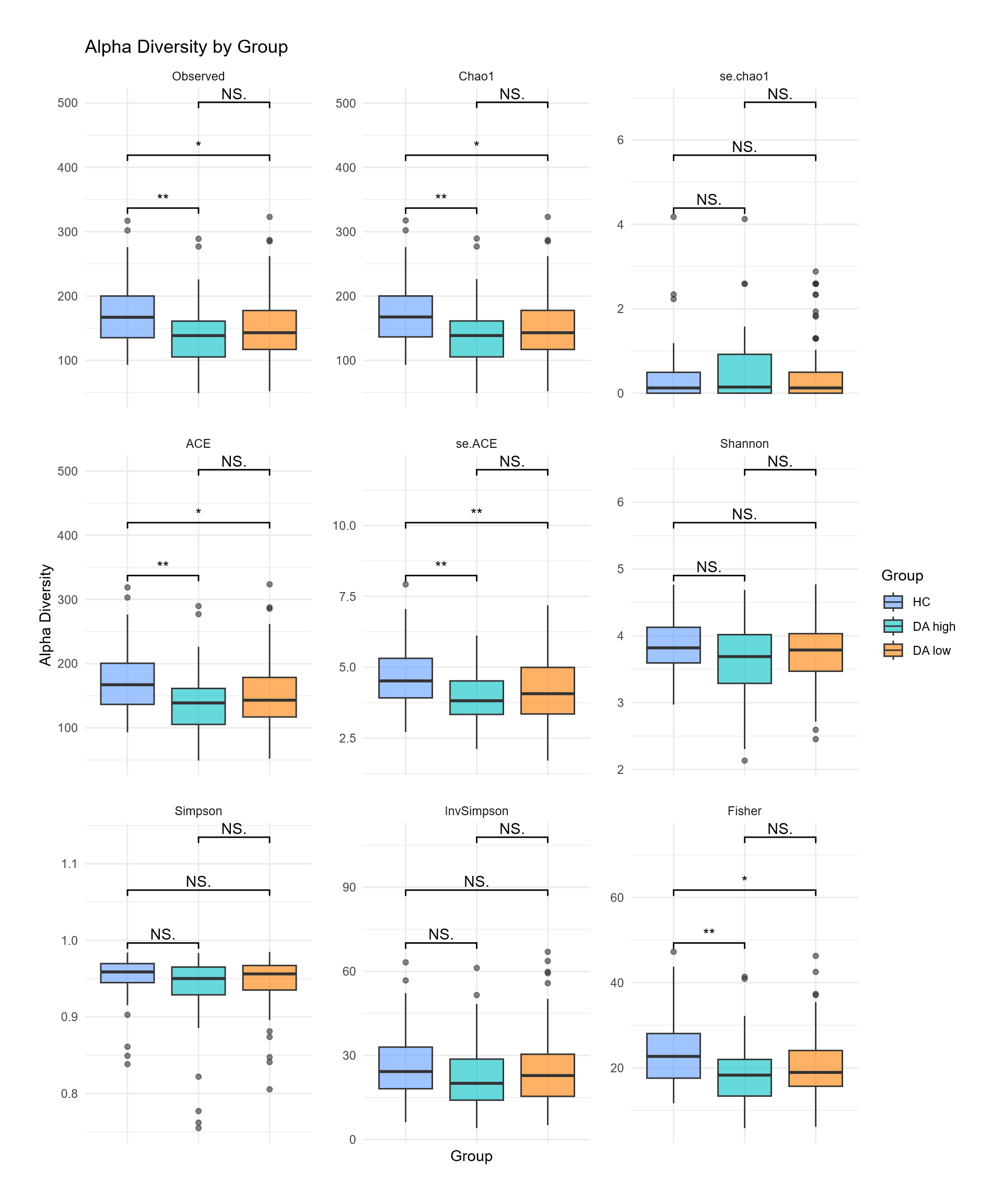


**Figure S4.** Alpha diversity of gut microbiota according to SLE disease activity. Boxplots display alpha diversity metrics for healthy controls (HC, *n* = 50, blue), SLE patients with high disease activity (DA high, *n* = 42, turquoise), and those with low disease activity (DA low, n = 115, orange). Nine commonly used alpha diversity indices are presented, including Observed species, Chao1, ACE, Shannon, Simpson, inverse Simpson, and Fisher's alpha. These indices represent various aspects of microbial community richness and evenness. Each boxplot shows the median, interquartile range (IQR), whiskers (1.5 × IQR), and outliers. Statistical significance was assessed using the Wilcoxon rank-sum test for pairwise comparisons. Significance levels are indicated as follows; NS: not significant, *: *p* < 0.05, **: *p* < 0.01, ***: *p* < 0.001.

.


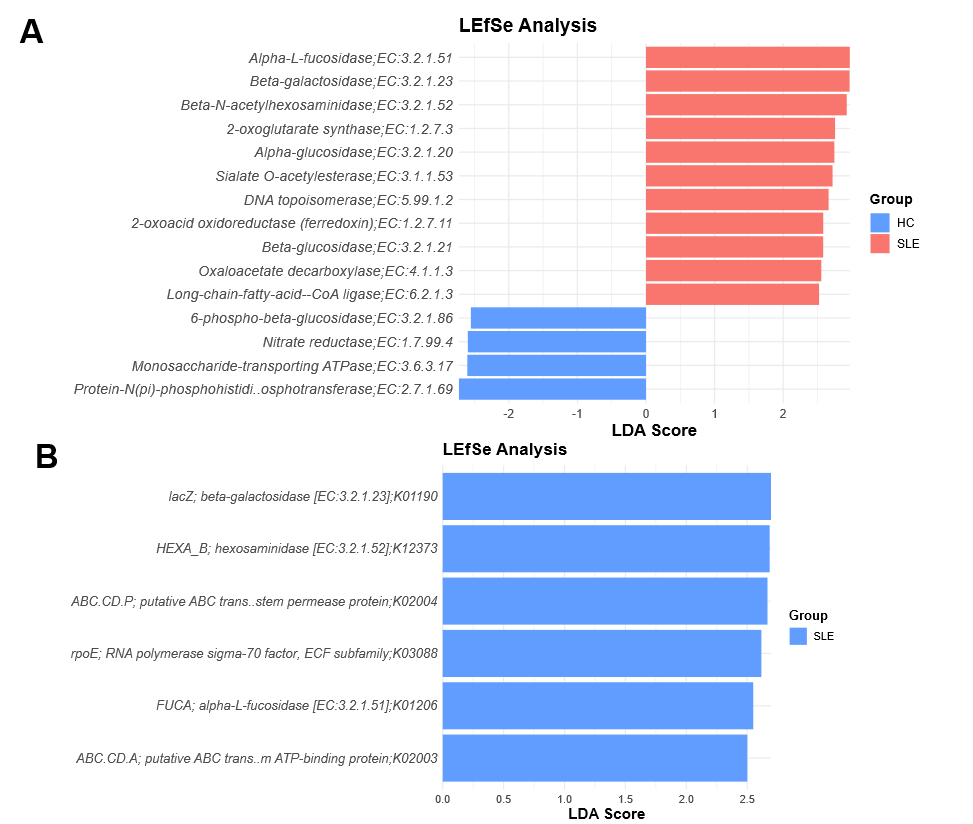


**Figure S5**. Functional prediction of microbial communities using PICRUSt2 in SLE and healthy controls. LEfSe analysis was applied to infer functionally distinct microbial pathways between SLE patients (*n* = 157) and healthy controls (HC, *n* = 50) based on PICRUSt2-predicted metagenomes. (**A**) The bar plot displays differentially abundant microbial enzyme functions based on Enzyme Commission (EC) numbers with LDA scores ≥ 2.0. (**B**) LEfSe analysis at the KEGG Orthology (KO) level shows significantly enriched functional genes in the SLE group. Functional comparisons were conducted using Welch’s t-test followed by FDR correction (*q* ≤ 0.05).
